# Circulating Metabolites in Relation to the Kidney Allograft Function in Posttransplant Patients

**DOI:** 10.3390/metabo12070661

**Published:** 2022-07-18

**Authors:** Eva Baranovicova, Matej Vnucak, Karol Granak, Jan Lehotsky, Nina Kadasova, Juraj Miklusica, Ivana Dedinska

**Affiliations:** 1 Biomedical Centre BioMed, Jessenius Faculty of Medicine, Comenius University in Bratislava, Mala Hora 4, 036 01 Martin, Slovakia; eva.baranovicova@uniba.sk; 2 Transplant Center, I. Internal Department, University Hospital Martin, Jessenius Faculty of Medicine, Comenius University in Bratislava, Mala Hora 4, 036 01 Martin, Slovakia; matej.vnucak@uniba.sk (M.V.); karol.granak@uniba.sk (K.G.); 3 Department of Medical Biochemistry, Jessenius Faculty of Medicine, Comenius University in Bratislava, Mala Hora 4, 036 01 Martin, Slovakia; jan.lehotsky@uniba.sk; 4 Department of Biophysics, Faculty of Science, Palacky University, 17. listopadu 12, 771 46 Olomouc, Czech Republic; nina.kadasova@gmail.com; 5 Department of General, Visceral and Transplant Surgery, University Hospital Martin, Jessenius Faculty of Medicine, Comenius University in Bratislava, Mala Hora 4, 036 01 Martin, Slovakia; juraj.miklusica@uniba.sk

**Keywords:** NMR plasma metabolomics, kidney transplantation, allograft function

## Abstract

End-stage kidney disease is preferably treated by kidney transplantation. The suboptimal function of the allograft often results in misbalances in kidney-controlled processes and requires long-term monitoring of allograft function and viability. As the kidneys are organs with a very high metabolomic rate, a metabolomics approach is suitable to describe systematic changes in post-transplant patients and has great potential for monitoring allograft function, which has not been described yet. In this study, we used blood plasma samples from 55 patients after primary kidney transplantation identically treated with immunosuppressants with follow-up 50 months in the mean after surgery and evaluated relative levels of basal plasma metabolites detectable by NMR spectroscopy. We were looking for the correlations between circulating metabolites levels and allograft performance and allograft rejection features. Our results imply a quantitative relationship between restricted renal function, insufficient hydroxylation of phenylalanine to tyrosine, lowered renal glutamine utilization, shifted nitrogen balance, and other alterations that are not related exclusively to the metabolism of the kidney. No link between allograft function and energy metabolism can be concluded, as no changes were found for glucose, glycolytic intermediates, and 3-hydroxybutyrate as a ketone body representative. The observed changes are to be seen as a superposition of changes in the comprehensive inter-organ metabolic exchange, when the restricted function of one organ may induce compensatory effects or cause secondary alterations. Particular differences in plasma metabolite levels in patients with acute cellular and antibody-mediated allograft rejection were considered rather to be related to the loss of kidney function than to the molecular mechanism of graft rejection since they largely follow the alterations observed by restricted allograft function. In the end, we showed using a simple mathematical model, multilinear regression, that the basal plasmatic metabolites correlated with allograft function expressed by the level of glomerular filtration rate (with creatinine: *p*-value = 4.0 × 10^−26^ and r = 0.94, without creatinine: *p*-value = 3.2 × 10^−22^ and r = 0.91) make the noninvasive estimation of the allograft function feasible.

## 1. Introduction

Kidneys are the organs with the second-highest metabolic rate, and restricted renal function results in misbalances in kidney-controlled/participating processes, such as the maintenance of acid-base equilibrium, electrolyte and fluid balances, the regulation of hematopoiesis, and the excretion of waste products [1], but also the exchange of nitrogenous metabolites between organs [2,3]. End-stage kidney diseases are preferably treated by kidney transplantation to replenish all the processes restricted by kidney failure with a maximal effort to improve the survival and long-term outcome of patients. A kidney from a living donor usually functions immediately; however, its performance is often suboptimal and should be monitored over the whole post-transplantation period. Therefore, the priority of the transplant expert community is to identify new noninvasive features that correlate with or predict graft function, which would benefit the patients.

In the field of renal transplantation, metabolomic studies have reported interesting results. Adequate metabolic recovery was recognized as a critical determinant of outcome after kidney transplantation [4]. The metabolomic profiling of urine enabled the non-invasive estimation of the recovery process of kidney-transplanted patients [5], offered a noninvasive means of diagnosing and predicting acute cellular rejection in the human kidney allograft [6], and allowed the monitoring of kidney graft recovery to identify patients who are not progressing within the prospective normal range [7].

Only sparse information can be found about the metabolomic changes in blood plasma in relation to allograft function. Circulation serves as the pool of low molecular species ensuring inter-organ metabolic communication and exchange. With an unreliably working kidney, the organism becomes metabolically challenged, and the imbalance may lead to compensatory effects of the other organs as well as secondary damage. The worsening of renal function is clinically determined by two basal approaches—by increasing serum creatinine (Cre) and decreasing eGFR (estimated glomerular filtration rate), which serve also as a quantitative indicator of allograft viability and performance. In this study, we used both mentioned parameters to assess allograft function, and we looked for how the deteriorating condition of the kidneys in post-transplant patients affects levels of plasma metabolites, which have not been explored yet. To achieve as homogeneous a group as possible, we selectively included patients after primary kidney transplantation, identically treated with immunosuppressants and more than 5 years after transplantation in the mean. We also attempted to use the metabolomic profile in the blood to predict allograft function and allograft rejection, which could be of great use as a noninvasive method in clinical practice.

## 2. Materials and Methods

### 2.1. Patients

The presented analysis consisted of a total number of 55 adult patients (Caucasians) after the primary transplantation of a kidney from a standard criteria brain-dead donor (SCD), who underwent the transplantation in the Transplant Centre Martin, Slovakia. We determined the following parameters of all patients included in the monitoring: age at the time of transplantation, sex, presence of biopsy-proven acute rejection (acute cellular rejection (ACR), acute antibody-mediated rejection (AMR)), proteinuria (standard 24 h urine protein test) and finally eGFR-estimated glomerular filtration rate (according to Chronic Kidney Disease Epidemiology Collaboration (CKD-EPI) formula). Acute rejection was confirmed by graft biopsy and classified according to the Banff classification 2019. Donor-specific antibodies were determined by the LUMINEX methodology (positivity was stipulated at ≥500 MFI). Standard immunosuppression was identical in the whole group, namely tacrolimus (TAC, Advagraf), mycophenolate sodium (Myfortic) and Prednisone. The group of patients was homogenous in terms of immunosuppression-average levels of TAC, and daily doses of mycophenolate sodium (Myfortic) and Prednisone were similar (with no significant difference between monitored groups). The average follow-up was 68.4 (SD = 50.7) months after kidney transplantation.

Inclusion criteria were as follows:

- Age > 18 years,
- No active infection,
- Immunosuppression consisted of TAC, MPA, and Prednisone,
- Primary kidney transplantation from SCD.

Exclusion criteria were as follows:

- Age < 18 years,
- Lost to follow up,
- Active infection,
- Kidney transplantation from an extended criteria donor (ECD) or living kidney donor,
- Kidney transplantation in the last 6 months.

A donor with extended criteria means a donor according to the definition of ECD codified in 2002: donors over the age of 60 years without co-morbidities or donors over the age of 50 years with at least two co-morbidities that include blood hypertension, death from cerebrovascular accident, or terminal stage. At the time of blood collection, all patients had clinically determined Cre, eGFR, and proteinuria levels. The stage was assigned by eGFR values according to the generally accepted CKD-EPI formula and KDIGO CKD classification (stage 1 for eGFR 90 and higher, stage 2 for eGFR 60–89, stage 3 for eGFR 30–59, stage 4 for eGFR 15–29, and stage 5 for eGFR less than 15). Patients’ characteristics are summarized in Table 1 and Table 2.

### 2.2. Samples Preparation

Blood was collected in EDTA-coated tubes and, within 1 h after collection, centrifuged to plasma at 4 °C, 2000 rpm (380 *g*-force) for 20 min. The samples were stored at −80 °C until use. Plasma denaturation was carried out according to Gowda et al. [8]: 600 µL of methanol was added to 300 µL of blood plasma, and the mixture was vortexed and frozen at −24 °C for 20 min. After subsequent centrifugation at 14,000 rpm (14,800× *g*-force) for 30 min, 700 µL of supernatant was taken, dried out and stored at −80 °C. Before NMR measurement, the dried matter was mixed with 100 µL of stock solution (consisting of phosphate buffer 200 mmol/L pH 7.4, 0.30 mmol/l TSP-d_4_ (trimethylsilylpropionic acid-d4) as a chemical shift reference in deuterated water) and 500 µL of deuterated water. Finally, 550 µL of the final mixture was transferred into a 5 mm NMR tube.

### 2.3. Data Acquisition

NMR data were acquired on 600 MHz NMR spectrometer Avance III from Bruker equipped with TCI CryoProbe at T = 310 K. Initial settings (field shimming, receiver gain, water suppression frequency) were carried out on an independent sample and adopted for measurements. After preparation, samples were stored in a Sample Jet automatic machine for not more than 2 h and cooled at approximately 5 °C. Before measurement, each sample was preheated on the 310 K for 5 min. An exponential noise filter was used to introduce 0.3 Hz line broadening before Fourier transform. All data were once zero-filled. Samples were randomly ordered for acquisition. 

We modified standard profiling protocols from Bruker as follows: noesy with presaturation (noesygppr1d): FID size 64k, dummy scans 4, number of scans: 64, spectral width 20.4750 ppm; profiling cpmg (cpmgpr1d, L4 = 126, d20 = 3 ms): number of scans: 256, spectral width 20.4750 ppm. For 15 randomly chosen samples, 2D spectra were measured: cosy with presaturation (cosygpprqf): FID size 4 k, dummy scans 8, number of scans 16, spectral width 16.0125 ppm; homonuclear J-resolved (jresgpprqf): FID size 8 k, dummy scans 16, number of scans 32. Samples were randomly ordered for acquisition. For samples, we kept the half-width of the TSP-d_4_ signal under 1.0 Hz. All experiments were conducted with a relaxation delay of 4 s.

Spectra were solved using a human metabolomic database (www.hmda.ca) (accessed on 15 March 2022) [9], Chenomix software (free trial version), an internal metabolite database, and by researching in metabolomic literature [8]. The proton NMR chemical shifts are reported relative to the TSP-d_4_ signal assigned a chemical shift of 0.000 ppm. The peak multiplicities were confirmed in J-resolved spectra, and homonuclear cross-peaks were confirmed in 2D cosy spectra. Peaks assignments are listed in Supplement Table S1.

All spectra were binned to bins of the size of 0.001 ppm. No further normalization method was applied to the data, as we took exactly the same amount of blood plasma for measurements. Then, intensities of selected bins were summed only for spectra subregions with only one metabolite assigned or minimally affected by other co-metabolites. Metabolites showing weak intensive peaks or strong peak overlap were excluded from the evaluation. The obtained values were used as relative concentrations of particular metabolites in a given sample.

### 2.4. Data Processing

Pearson’s correlation was used to determine the linear relations between relative levels of blood plasma metabolites determined by the NMR method and the clinically determined plasma creatinine level (Cre), eGFR value, and level of proteinuria within the patients’ group. Calculations were carried out in OriginPro 2018b (v.9.5, OriginLab, Northampton, MA, USA) and Matlab (v. 2015b, Mathworks, Natick, MA, USA). A *p*-value of 0.05 was used as a threshold to claim significance. The null hypothesis of equality of population medians among stages and groups with/without rejection was tested by the non-parametrical Kruskal–Wallis test for multiple comparisons with Dun’s post hoc test for pairwise comparison using an online tool [10]. Both tests are designed for a non-normal distribution, which was assumed since the normality cannot be reliably tested in groups of size under 30. Stages 1 and 5 were excluded from these tests as they had only one and two representatives. Box plots were used to explore scaled metabolites intensities. For the discriminatory analysis of binary systems, we employed a cross-validated Random Forest discriminatory algorithm, included in the online tool Metaboanalyst [11].

Note that, in this work, we use the common labeling of BCAAs for branched-chain amino acids—leucine, isoleucine, and valine—and BCKAs for their 2-oxoderivates, branched-chain keto acids—ketoleucine (2-oxoisocaproate), ketosioleucine (3-methyl-2-oxopentanonate), and ketovaline (2-oxoisovalerate)—as well as mentioning the trivial names of BCKAs that better evoke their origin.

## 3. Results

Relative concentrations of plasma metabolites determined by NMR—alanine, phenylalanine, glutamine, proline and histidine—correlated significantly with clinically determined serum creatinine levels. All these metabolites except alanine but together with acetate, citrate, and branched-chain keto-acids (BCKAs) (boundary *p*-values 0.02, 0.05 and 0.08) correlated with eGFR, and together with alanine also with the stage related to the kidney function. Only BCKAs plasma levels correlated with the level of proteinuria, which is generally recognized as a marker of the severity of chronic kidney disease and as a predictor of a future decline in glomerular filtration rate. The details are summarized in Table 3. No significant correlations were found for the metabolites lactate, BCAAs, glucose, pyruvate, 3-hydroxybutyrate, and creatine. The correlations of creatinine levels determined by NMR were calculated, but they were excluded from the discussion since we operated with the clinically determined serum creatinine level (Cre) as one of the key parameters determining kidney function. The mutual relation of creatinine levels by both methods was of the *p*-value of 3.4 × 10^−15^ (Table 3). 

We performed a multilinear regression analysis where it was of interest if a linear combination of relative plasma levels (as a very intuitive and simple regression tool) of selected metabolites could improve the correlations with eGFR and Cre. For the regression model, we used all determined plasma metabolites with and without creatinine as independent variables. The predicted vs. measured eGFR values correlated as follows: with creatinine: *p*-value = 4.0 × 10^−26^ and r = 0.94, without creatinine: *p*-value = 3.2 × 10^−22^ and r = 0.91. The predicted vs. measured Cre plasma level correlated as follows: with creatinine: *p*-value 2.1 × 10^−50^ and r = 0.99, and without creatinine: *p*-value = 5.3 × 10^−25^, r = 0.93.

**Figure 1:**
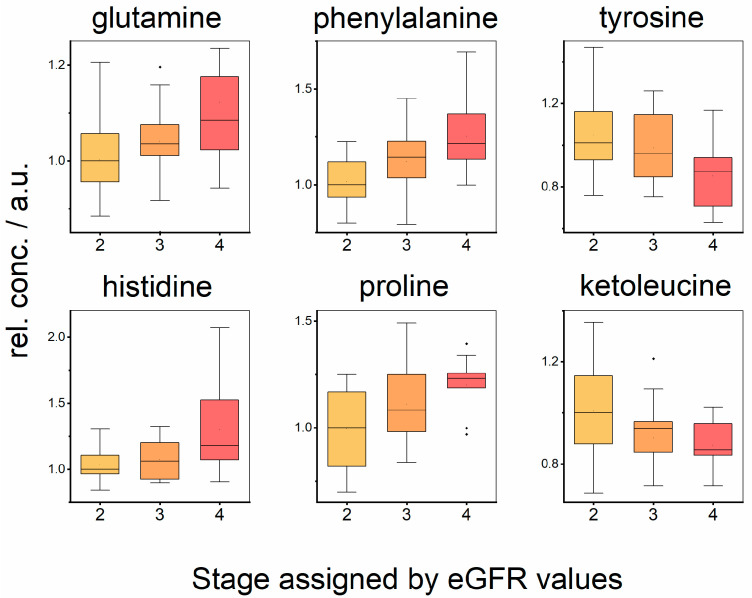
Relative concentrations of metabolites in blood plasma in patients showing kidney function in Stages 2, 3, and 4.

For metabolites showing strong relations to allograft function, we compared relative plasma levels for clinical stages (Table 4, Figure 1; for detailed determination of stages, please see Section 2). The results are in agreement with those expected from correlations in Table 3, as this is another kind of evaluation of the same data.

**Figure 2:**
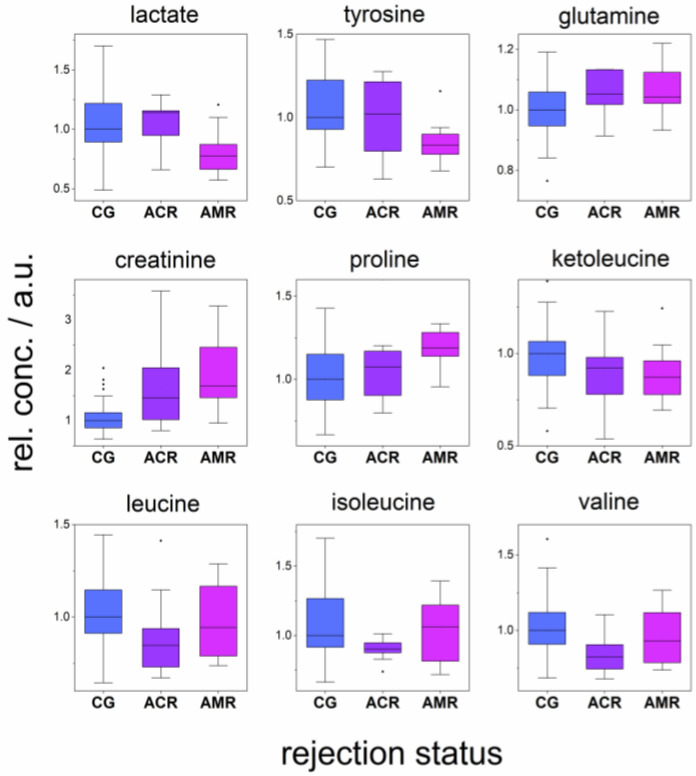
Relative concentrations of metabolites in blood plasma in patients without signs of rejection (CG), with acute cellular rejection (ACR), and acute antibody-mediated rejection (AMR).

The association between allograft rejection and the levels of plasma metabolites was assessed by the comparison of the relative plasma concentrations among patients without rejection (CG) and with acute cellular rejection (ACR) and acute antibody-mediated rejection (AMR). The metabolites levels found to be significantly different (*p*-value under 0.05) are listed in Table 5. Boundary significant differences in plasma BCKAs levels were observed for the comparison CG > AMR (*p*-values in the range 0.08–0.14; ketoleucine as an example is included in Figure 2). For other metabolites, no significant changes were found. 

**Figure 3:**
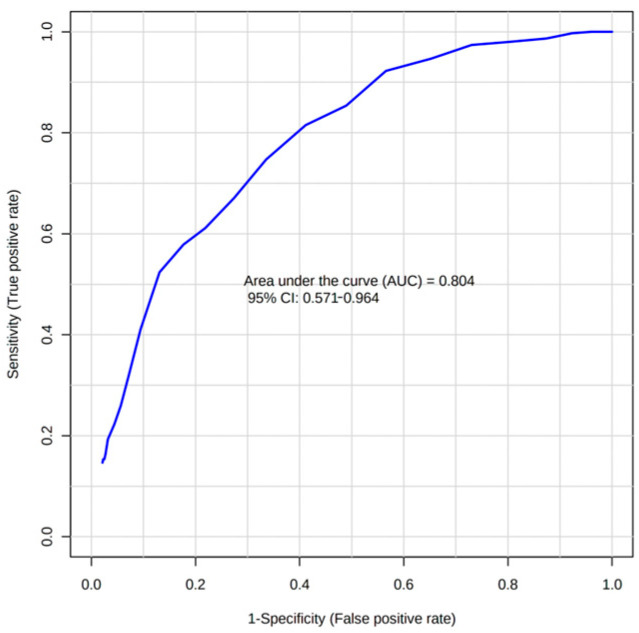
ROC curve derived from Random Forest discriminatory algorithm for binary system CG–AMR; as input data, the relative concentrations of plasma metabolites were used.

To assess the feasibility of plasma metabolites to predict graft rejection, we employed a discriminatory cross-validated Random Forrest (RF) algorithm. As input variables, relative concentrations of plasma metabolites were used. The parameter expressing the discrimination performance was the area under the curve (AUC), derived from the receiver operator characteristic curve (ROC). When discriminating CG against ACR, we obtained the AUC value of 0.49. Classification for the binary system CG–AMR was performed with AUC 0.80 when creatinine was included (Figure 3), where the metabolites creatinine, lactate, proline, tyrosine, and ketoleucine were of the highest importance. When creatinine was excluded, a weaker discriminatory effect was achieved with an AUC of 0.68. The discriminatory performance for the system ACR–AMR was very weak, with an AUC of 0.59.

## 4. Discussion

### 4.1. Alterations in Plasma Metabolites

One of the unique metabolic functions of the kidney is renal phenylalanine hydroxylation to tyrosine, which accounts for approximately 50% of whole-body phenylalanine hydroxylation [1]. Moller et al. demonstrated that the kidney is the major donor of tyrosine to the systemic circulation by the conversion of phenylalanine to tyrosine [12], and the kidneys alone would be capable of producing all the tyrosine needed by the body. In previous studies, it was observed that chronic renal failure leads to an impairment of whole-body phenylalanine hydroxylation [13], and tyrosine deficiency was detected also in end-stage renal disease [14]. In this study, we observed increased circulating plasma phenylalanine levels with worsening allograft function (Table 3) in patients after kidney transplantation. This observation was confirmed also in the intergroup comparison (Figure 1), where plasma phenylalanine levels elevated with the stage of allograft insufficiency. Additionally, we obtained the opposite correlations for tyrosine, which decreased with worsening renal function; however, only the boundary was statistically significant (*p*-value 0.05 with Cre and 0.16 with eGFR, Table 3). We observed also decreased plasma tyrosine levels with worsening allograft stage (Figure 1). Remarkable tyrosine plasma depletion was also observed in AMR patients against ACR and CG (Figure 2), which is probably linked with differences in graft function (Table 2). Elevated plasma phenylalanine and lowered plasma tyrosine are suggested to decrease the renal hydroxylation of phenylalanine to tyrosine in the case of a not fully functional allograft. These circumstances may have additional important clinical implications for patients, as they are consequently challenged with the higher risk of phenylalanine overloading and tyrosine deficiency, which could lead to the limited availability of the tyrosine products and neurotransmitters dopamine, catecholamines, and thyroid hormones, and thus cause an additional burden on the organism. 

Glutamine is one of the most abundant amino acids in plasma. It belongs to the non-essential amino acids; however, under conditions where de novo synthesis does not fulfill the body’s requirements, it is considered as conditionally essential. Besides being a building block for proteins, it serves as essential fuel for all fast-dividing cells, including immunocompetent cells whose proliferation is crucially dependent on glutamine availability [15,16,17]. Renal glutamine uptake is quantitatively as important as utilization by the gut or immune system, and it represents 10–15% of daily whole-body glutamine turnover [1]. In our work, worsening graft function was accompanied by increasing plasma glutamine levels (Table 3). In the kidneys, each molecule of glutamine is metabolized by a series of reactions to ultimately form two bicarbonate anions that are reabsorbed into the bloodstream and two ammonia cations that are excreted in the urine [18]. Renal ammonia excretion is approximately 50% of total ammonia production, and 50% is returned to the renal vein [19]. Both ions are an important part of the plasma acid–base balance buffering system that is physiologically controlled by stimulating/reducing, as appropriate, glutamine metabolism in the kidney. During metabolic acidosis, commonly observed in patients with chronic kidney disease [20], and after kidney transplantation [21], ammonia excretion is impaired, tubular bicarbonate reabsorption is reduced, and renal bicarbonate production is insufficient [22]. Subsequently, skeletal protein muscle degradation is stimulated, and hepatic glutamine synthesis increases as a consequence of increased hepatic amino acid metabolization [23]. The observed increased glutamine levels with worsening allograft function are suggestive of insufficient glutamine utilization selectively in the kidneys. This partial conclusion is supported by the results from the pioneer study by Tizianelo et al. [24], who showed decreased glutamine uptake by the kidney in patients with chronic renal insufficiency. Restricted glutamine utilization by the kidney can—but must not, due to the other compensatory mechanisms—potentially support the development of metabolic acidosis. Patients included in our work had metabolic acidosis compensated by bicarbonate or exhibited normal Astrup values without necessary compensation, so in the group included in the study, insufficient glutamine utilization did not ultimately lead to disturbed acid–basic balance. 

The post-transplant patients included in our study with acute cellular rejection and acute antibody-mediated rejections showed glutamine plasma levels almost equal to each other and higher in comparison with patients without features of rejection (Figure 2). The elevated glutamine plasma level in ACR and AMR patients in comparison to CG is likely linked with worsening of allograft function, as discussed above. AMR is more often associated with worse graft function than ACR, and our AMR patients showed also worse Cre and eGFR values in comparison to ACR (Table 2); nevertheless, the expected difference in plasma glutamine level between these two groups was not found. We suppose that the glutamine plasma level in AMR increased due to the restricted kidney function, which could be in parallel partially exhausted by the elevated activation of T-cells, resulting in glutamine depletion in AMR patients to the level of ACR (Figure 2). 

Taken together, we assume that the plasma glutamine level in patients after kidney transplantation is not straightforwardly associated with only one process, but it reflects the combination of three fundamental processes: decreased glutamine utilization by the kidney, balancing glutamine production by the liver or muscles, and accelerated glutamine consumption by an increasingly activated immune system.

Relative plasma concentrations of BCKAs, ketoacids produced by deamination from BCAAs, were positively correlated with eGFR (*p*-values 0.02, 0.05, and 0.08) and negatively with proteinuria (all *p*-values were significant). In another statistical evaluation, ketoacids visibly decreased in the order CG > ACR > AMR (Figure 3 for ketoleucine), although cut-off the comparison CG > AMR was only slightly above the level of significance (*p*-values in the range 0.08–0.14). It seems that, in parallel with worsening graft function, the organism is lacking BCKAs that can neutralize the excessive nitrogen residues through transamination to BCAAs. This property, as well as the fact that ketoacids allow the preservation of nutritional status in the time of low protein intake, makes BCKAs potential adepts for supplementation in patients with worsening function of the allograft. The positive effect of ketoacid supplementation is already well established in experimental models [25] and also in humans, where ketoacid supplementation with a low-protein diet slowed down renal function deterioration [26]. A shifted nitrogen balance in the time of impaired graft function is manifested also in increasing plasma alanine levels with increasing Cre values. Alanine is released in large amounts from skeletal muscles for hepatic gluconeogenesis. The glucose–alanine cycle is critical for nitrogen metabolism and provides a nontoxic alternative for transferring amino groups derived from muscle amino-acid catabolism to the liver. 

**Figure 4:**
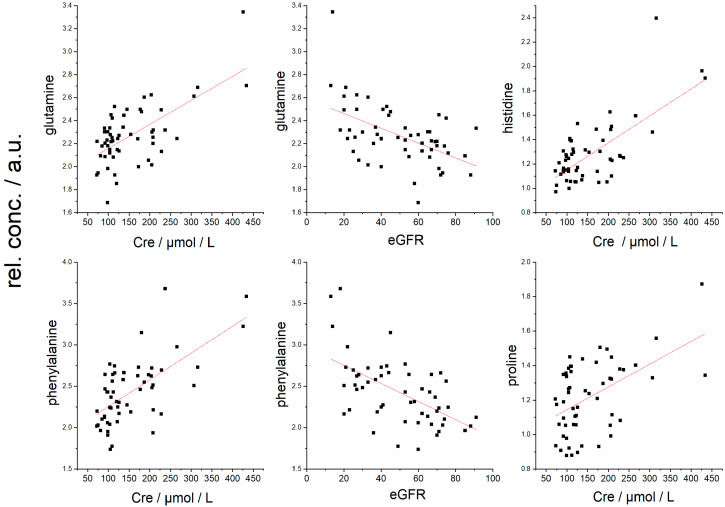
Plasma levels of glutamine, phenylalanine, histidine, and proline in relation to eGFR, and plasma levels of histidine and proline in the relation to serum creatinine—Cre.

The next metabolite that presented a relation to graft function was histidine, whose plasma levels increased with Cre and decreased with eGFR (Table 3, Figure 4). Histidine cannot be synthesized de novo in humans, and its main source remains the diet. Studies have found that L-histidine exhibits antioxidant capabilities, such as scavenging free radicals and chelating divalent metal ions [27,28]. Besides that, histidine can suppress the accumulation of IL-6 and TNF-α mRNA in a dose-dependent manner and could directly affect the regulation of pro-inflammatory cytokines [29], which were found to be elevated in the renal posttransplantation study, where the serum levels of both IL-6 and TNF-α were increased [30]. However, the immune response is known to be controlled also by histamine, an amine produced exclusively by histidine decarboxylation. The pleiotropic actions of histamine due to the different natures of its receptors allow it to exert broad and opposing effects on the immune system [31]—histamine balances important inflammatory reactions as well as immunomodulation [31]. A direct relationship between circulating histidine and restricted renal function has not been fully described yet. Probably, two main factors contribute to histidine elevation in blood plasma: its decreased utilization and accelerated protein catabolism. Given the strong relation of plasma histidine to allograft function (Table 3) and the fact that both histidine and its main metabolic product histamine participate in immune processes, their role could be worthy of deeper analysis and examination in the complex immune response after transplantation. 

The circulating proline in plasma is derived from the diet, intra- and extra-cellular protein degradation, and endogenous synthesis from glutamate or ornithine. Proline was found previously to be increased in blood plasma in patients with chronic renal insufficiency [24], indicating a linkage between increased plasma proline and the impairment of renal function. In parallel, we observed the correlation between plasma proline level and deteriorating graft function in kidney post-transplant patients. This trend was confirmed also by the other results, where proline increased in the order CG < ACR < AMR (Table 5, Figure 2), which is also indirectly linked with impaired graft function. Increasing plasma levels with loss of graft function were observed in our study also for acetate and citrate. Acetate confers numerous metabolic functions, including energy production, lipid synthesis, and protein acetylation. Citrate is a key metabolite of the energy-gaining Krebs cycle, it is freely filtered at the kidney glomerulus, and then in the amount of 65–90% is reabsorbed in the proximal tubule, leaving about 10–35% of the filtered excreted into the urine [32,33]. It is also metabolic fuel for the kidney and an endogenous inhibitor of calcium kidney stones [33]. Interestingly, it can be renally metabolized to HCO_3_^−^ and consequently represents a potential base, indicating its possible but not extensively researched role in the acid–base balance [33].

In this study, we did not observe any tendency in the alterations in energy metabolism with the loss of allograft function, as no correlations were found for glucose, glycolytic intermediates pyruvate and lactate, and another energy substrate—ketone body representative 3-hydroxybutyrate. Insulin insufficiency would be manifested in an increase in BCAAs, which was not observed, as they are (not causative) markers of loss of insulin action [34]. Leucine, isoleucine, and valine showed an identical decrease in patients with ACR when compared to CG or AMR (Table 5, Figure 2). It is known that BCAAs are essential for lymphocyte responsiveness and are necessary to support other immune cell functions [35]; it is likely that the essentiality of BCAAs for immune cells is related to protein synthesis. BCAAs also act as donors of nitrogen and carbon skeleton for the synthesis of other amino acids, such as glutamine. However, the biochemical mechanism that could lead to the selective depletion of BCAAs in ACR conditions cannot be simply derived from the current literature. Lastly, a decrease in blood plasma lactate level was also observed in AMR patients against both ACR and CG. 

### 4.2. Circulating Metabolites as Predictors of Allograft Rejection

For the classification of patients divided into subgroups by allograft rejection features, the Random Forest discriminatory algorithm was employed, and the performance was evaluated by the AUC value. To clarify, if the AUC equals 1, the system can be discriminated with 100% sensitivity and specificity; if the AUC equals 0.5, the data are of no practical utility as the probability of correct classification is 50%. The RF algorithm obtained an AUC of 0.8 for the binary system CG–AMR (Figure 3). The performance became weaker after the exclusion of creatinine from variables, resulting in an AUC of 0.68. Other discriminations between groups were poor. We assume that the differences between CG, ACR, and AMR groups, as discussed above, are rather related to the loss of kidney function than to the molecular mechanism of graft rejection since the differences among groups largely follow the differences obtained by different allograft functions. The only exception may be alterations in plasma glutamine, as already discussed above. These results indicate that the differences in the levels of basal plasma metabolites are not strong enough to predict allograft rejection, and the allograft function is the key factor determining the success of statistical discrimination between groups. 

### 4.3. Circulating Metabolites as Predictors of Allograft Function

**Figure 5:**
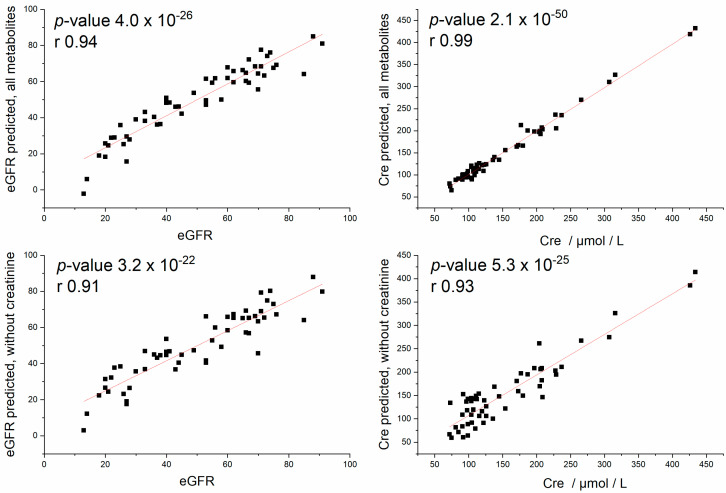
Results from Pearson’s correlation following multilinear regression analysis, where relative plasma concentrations were used as independent variables; predicted vs. measured eGFR (with creatinine: *p*-value = 4.0 × 10^−26^ and r = 0.94, without creatinine: *p*-value = 3.2 × 10^−22^ and r = 0.91) and predicted vs. measured Cre plasma level (with creatinine: *p*-value 2.1 × 10^−50^ and r = 0.99, and without creatinine: *p*-value = 5.3 × 10^−25^, r = 0.93).

The search for features that could help to estimate graft function besides the widely used eGFR or Cre level is challenging. The particular relation of some individual metabolites to allograft function expressed by eGFR or Cre observed in this study is discussed above. In this part, we wanted to verify if better relations could be achieved by combinations of metabolites. For this, we employed probably the easiest and most simply understandable multilinear regression model. The great potential of this evaluation method lies in (i) the very good availability of blood plasma, (ii) the fact that the metabolites included in the model are easily detectable by many analytical methods, and (iii) the simple mathematical model used. We compared two models: the first model with relative levels of all plasma metabolites determined by NMR (including creatinine) and the second model without NMR to determine creatinine level. The Cre value should be considered in this section as a quantitative parameter expressing graft function. We examined if the levels of basal plasma metabolites can predict the loss of graft function expressed in eGFR or Cre, respectively. We gained a very promising correlation between predicted and clinically determined eGFR values and an almost ideal correlation between predicted and clinically determined Cre, when using the model with all metabolites, including NMR-determined creatinine (Figure 5). To suppress the natural relations, we ran the prediction without NMR-determined creatinine. IN this way, we obtained promising correlations coefficients: r = 0.91 for eGFR and r = 0.93 for Cre. The presence of creatinine in the model has a beneficial but not essential role in prediction accuracy (Figure 5). The observed relation is very promising; however, we suppose that it is hard to achieve it as ideal, since the metabolomic state is not a reflection of only the kidney function and related metabolism but is also formed by other contributions not related to renal performance (metabolic, neurological, cardiovascular, gastrointestinal, and other diseases). 

## 5. Conclusions

In this work, we described the relations between kidney allograft function and the levels of basal plasma metabolites in the group of 55 patients after primary kidney transplantation. We discussed restricted phenylalanine to tyrosine hydroxylation by a weakly working allograft, decreased glutamine utilization by the kidney, balancing glutamine production by the liver or muscles, and accelerated glutamine consumption by an increasingly activated immune system as well as shifted nitrogen balance. It seems that allograft function is not linked with energy metabolism, as no changes were found for glucose, glycolytic intermediates, and 3-hydroxybutyrate as a ketone body representative. We concluded that the observed metabolomic changes in plasma are not related exclusively to the sole metabolomic role of the kidney, but the observed relations between metabolomics plasma levels and the allograft function are the results of the complex mutual biochemical pathways in the comprehensive inter-organ metabolic exchange and communication when the restricted function of one organ induces compensatory effects or secondary injury.

The metabolomics alterations in blood plasma in patients with allograft rejection could be largely explained by the various extents of allograft function in these subgroups and only barely affected by the rejection mechanism. The observed changes were not strong enough to satisfactorily classify patients with ACR and AMR rejection. The best result was obtained in the discrimination of patients without rejection to AMR, which was performed with an AUC of 0.80—after the exclusion of creatinine, the AUC was 0.68. However, we showed that the basal plasmatic metabolites in combination manifested a very high correlation with eGFR (with creatinine: *p*-value = 4.0 × 10^−26^ and r = 0.94, without creatinine: *p*-value = 3.2 × 10^−22^ and r = 0.91), and the extent of metabolomics changes in blood plasma has high potential for the estimation of allograft function.

## Figures and Tables

**Table 1:** Characteristics of patients enrolled in the study by stage, mean values with standard deviation (SD).

Parameter	All	Stage 1	Stage 2	Stage 3	Stage 4	Stage 5
Sample size	55	1	21	21	10	2
Age/years (SD)	52.6 (13.8)	41	48.4 (13.6)	56.8 (13.7)	54.4 (12.7)	42, 48 (2 values)
Sex (F/M)	23/32	0/1	10/11	8/13	5/5	0/2
BMI	28.3 (6.1)	28.1	27.2 (4.7)	27.7 (5.7)	28.9 (5.4)	25.7, 51.2 (2 values)
Cre/µmol/L (SD)	154.6 (79.2)	91	98.3 (14.3)	148.5 (38.2)	237.0 (46.2)	426, 434 (2 values)
eGFR/mL/min/per 1.73 m^2^ (SD)	49.4 (20.8)	91	69.9 (7.1)	43.0 (8.9)	23.7 (2.9)	13, 14 (2 values)
Proteinuria level/g/ 24 h (SD)	0.847 (1.965)	0.126	0.286 (0.443)	0.985 (2.081)	0.6684 (0.788)	1.17, 11.024 (2 values)

**Table 2:** Characteristics of patients enrolled in the study by graft rejection, mean values with standard deviation (SD), patients without signs of rejection (CG), with acute cellular rejection (ACR), and with acute antibody-mediated rejection (AMR).

Parameter	CG	ACR	AMR
Sample size	35	10	10
Age/years (SD)	56.6 (16.1)	50.0 (12.7)	46.0 (14.2)
Gender (F/M)	14/21	4/6	5/5
BMI	28.6 (5.2)	29.5 (8.3)	26.1 (5.9)
Cre/µmol/L (SD)	123 (43.2)	185.5 (95.9)	234.2 (89.3)
eGFR/mL/min/per 1.73 m^2^ (SD)	58.0 (18.1)	40.6 (18.0)	28.3 (11.2)
Proteinuria level/g/24 h (SD)	0.365 (0.458)	1.222 (2.828)	2.155 (3.076)

**Table 3:** Pearson’s correlations of serum creatinine (Cre), eGFR, and proteinuria with relative metabolites level in blood plasma, determined by NMR spectroscopy, as well as the interrelationships among patients’ parameters, * significant correlation.

	Cre (µmol/L)		eGFR (mL/min/per 1.73 m^2^)		Proteinuria (g/24 h)	
	r	*p*-Value	r	*p*-Value	r	*p*-Value
Cre (µmol/L)	1	-	−0.83	2.9 × 10^−15^ *	0.47	0.00024 *
eGFR (mL/min/per 1.73 m^2^)	−0.83	2.9 × 10^−15^ *	1	-	−0.34	0.0089 *
Stage	0.84	6.6 × 10^−16^ *	−0.92	1.6 × 10^−23^ *	0.34	0.010 *
Proteinuria (g/24 h)	0.47	0.00024 *	−0.34	0.0089 *	1	-
Alanine	0.28	0.044 *	−0.11	0.40	0.02	0.86
Acetate	0.17	0.19	−0.29	0.029 *	0.06	0.64
Citrate	0.10	0.43	−0.29	0.031 *	−0.04	0.76
Phenylalanine	0.45	0.00044 *	−0.46	0.00030 *	−0.06	0.62
Tyrosine	−0.26	0.05	0.18	0.16	−0.20	0.12
Glutamine	0.48	0.00016 *	−0.39	0.0026 *	0	0.98
Ketoleucine	−0.14	0.27	0.30	0.02 *	−0.36	0.0055 *
Ketoisoleucine	−0.15	0.26	0.23	0.08	−0.28	0.033 *
Ketovaline	−0.20	0.12	0.26	0.05	−0.43	0.0010 *
Creatinine	0.98	0.00016 *	−0.83	3.4 × 10^−15^ *	0.42	0.0013 *
Proline	0.45	0.00049 *	−0.33	0.011 *	0.02	0.84
Histidine	0.66	3.6 × 10^−8^ *	−0.39	0.0031 *	0.13	0.32

**Table 4:** Evaluation of changes of relative metabolites levels in blood plasma in patients in Stages 2, 3, and 4, *p*-values obtained by Kruskal–Wallis test for multiple comparisons with post hoc Dun’s test for pairwise comparison for Stages 2, 3, and 4. The direction of changes is indicated in parenthesis, * significant difference.

Metabolite	Stage 2, 3, 4	Stage 2–3	Stage 2–4	Stage 3–4
Glutamine	0.035 * (2 < 3 < 4)	0.155 (2 < 3)	0.01 * (2 < 4)	0.17 (3 < 4)
Phenylalanine	0.0019 * (2 < 3 < 4)	0.019 * (2 < 3)	0.00072 * (2 < 4)	0.14 (3 < 4)
Tyrosine	0.033 * (2 > 3 > 4)	0.34 (2 > 3)	0.0091 * (2 > 4)	0.06 (3 > 4)
Histidine	0.085 (2 < 3 < 4)	0.57 (2 < 3)	0.028 * (2 < 4)	0.09 (3 < 4)
Proline	0.019 * (2 < 3 < 4)	0.08 (2 < 3)	0.0065 * (2 < 4)	0.16 (3 < 4)
Ketoleucine	0.028 * (2 > 3 > 4)	0.04 * (2 > 3)	0.015 * (2 > 4)	0.43 (3 > 4)
Ketoisoleucine	0.082 (2 > 3 > 4)	0.39 (2 > 3)	0.041 * (2 > 4)	0.043 * (3 > 4)
Ketovaline	0.142 (2 > 3 > 4)	0.41 (2 > 3)	0.047 * (2 > 4)	0.17 (3 > 4)

**Table 5:** Evaluation of changes of relative levels of plasma metabolites in patients without signs of rejection (CG), with acute cellular rejection (ACR), and acute antibody-mediated rejection (AMR), *p*-values obtained by Kruskal–Wallis test for multiple comparisons with post hoc Dun’s test for pairwise comparison. The direction of changes is indicated in parenthesis, * significant difference.

Metabolite	CG-ACR-AMR	CG-ACR	CG-AMR	ACR-AMR
Lactate	0.010 *	0.31	0.0096 * (CG > AMR)	0.0047 * (ACR > AMR)
Valine	0.014 *	0.0039 * (CG > ACR)	0.35	0.10
Leucine	0.021 *	0.0059 * (CG > ACR)	0.36	0.12
Isoleucine	0.11	0.035 * (CG >ACR)	0.64	0.17
Tyrosine	0.067	0.25	0.026 * (CG > AMR)	0.41
Glutamine	0.062	0.041 * (CG < ACR)	0.048 * (CG <AMR)	0.99
Creatinine	0.00016 *	0.0086 * (CG < ACR)	0.00019 * (CG < AMR)	0.44
Proline	0.015 *	0.53	0.0039 * (CG < AMR)	0.082

## Data Availability

The data presented in this study (NMR raw, evaluated, patients’ sharacteristic, statistical details) are available on request from the corresponding author. The data are not publicly available due to large volume of data (NMR spectra raw).

## Supplementary Materials

The following supporting information can be downloaded at https://www.mdpi.com/article/10.3390/metabo12070661/s1, Table S1: Chemical shifts (in ppm), J couplings (in Hz) and multiplicities (s—singlet, d—doublet, t—triplet, q—quartet, m—multiplet, dd—doublet of doublets, dq—doublet of quartets) for the pool of metabolites identified in blood plasma. Signals marked with # were not suitable for quantitative analyzes.
